# Is the Activity-Based Anorexia Model a Reliable Method of Presenting Peripheral Clinical Features of Anorexia Nervosa?

**DOI:** 10.3390/nu13082876

**Published:** 2021-08-21

**Authors:** Kamil Skowron, Magdalena Kurnik-Łucka, Michał Jurczyk, Veronika Aleksandrovych, Paulina Stach, Emil Dadański, Beata Kuśnierz-Cabala, Krzysztof Jasiński, Władysław P. Węglarz, Paulina Mazur, Piotr Podlasz, Krzysztof Wąsowicz, Krzysztof Gil

**Affiliations:** 1 Department of Pathophysiology, Faculty of Medicine, Jagiellonian University Medical College, 31-121 Krakow, Poland; magdalena.kurnik@uj.edu.pl (M.K.-Ł.); michal.jurczyk@uj.edu.pl (M.J.); v.aleksandrovych@uj.edu.pl (V.A.); paulina.stach.95@gmail.com (P.S.); emil.dadanski@gmail.com (E.D.); krzysztof.m.gil@uj.edu.pl (K.G.); 2 Department of Diagnostics, Faculty of Medicine, Jagiellonian University Medical College, 31-501 Krakow, Poland; beata.kusnierz-cabala@uj.edu.pl; 3 Institute of Nuclear Physics, Polish Academy of Sciences, 31-342 Krakow, Poland; krzysztof.jasinski@ifj.edu.pl (K.J.); wladyslaw.weglarz@ifj.edu.pl (W.P.W.); 4 Department of Medical Diagnostics, Faculty of Pharmacy, Jagiellonian University Medical College, 30-688 Krakow, Poland; paulina.pater@uj.edu.pl; 5 Department of Pathophysiology, Forensic Veterinary Medicine and Administration, Faculty of Veterinary Medicine, University of Warmia and Mazury in Olsztyn, 10-719 Olsztyn, Poland; piotr.podlasz@uwm.edu.pl (P.P.); wasowicz@uwm.edu.pl (K.W.)

**Keywords:** anorexia nervosa, activity-based anorexia, animal model, eating disorders, starvation, hyperactivity

## Abstract

Anorexia nervosa (AN) causes the highest number of deaths among all psychiatric disorders. Reduction in food intake and hyperactivity/increased anxiety observed in AN are also the core features of the activity-based anorexia animal model (ABA). Our aim was to assess how the acute ABA protocol mimics common AN complications, including gonadal and cardiovascular dysfunctions, depending on gender, age, and initial body weight, to form a comprehensive description of ABA as a reliable research tool. Wheel running, body weight, and food intake of adolescent female and male rats were monitored. Electrocardiography, heart rate variability, systolic blood pressure, and magnetic resonance imaging (MRI) measurements were performed. Immediately after euthanasia, tissue fragments and blood were collected for further analysis. Uterine weight was 2 times lower in ABA female rats, and ovarian tissue exhibited a reduced number of antral follicles and decreased expression of estrogen and progesterone receptors. Cardiovascular measurements revealed autonomic decompensation with prolongation of QRS complex and QT interval. The ABA model is a reliable research tool for presenting the breakdown of adaptation mechanisms observed in severe AN. Cardiac and hormonal features of ABA with underlying altered neuroendocrine pathways create a valid phenotype of a human disease.

## 1. Introduction

The concept of an activity-based anorexia (ABA) model (also referred to as exercise-induced anorexia or food restriction-induced hyperactivity) goes back to 1967, when Routtenberg and Kuznesof developed a food restriction protocol in conjunction with voluntary wheel access, which led to rapid weight loss, temporarily reduced food intake, and hyperactivity of rats [1]. In addition, isolation of rodents in individual cages adds social stress that accounts for the development of anorexia. Thus, a reduction of food intake/loss of appetite and hyperactivity/increasing anxiety as observed in human anorexia nervosa (AN) are also the core features of the ABA model. Numerous biological complications directly attributable to weight loss and malnutrition can be observed in AN patients. These complications affect almost every organ system and account for approximately half of all deaths in anorexia nervosa [2], with cardiac complications being the most common cause of death [3,4]. Arcelus et al. reported in a meta-analysis that the rate of death was 6 times greater in AN patients than in the general population, and age at assessment was defined as a significant predictor of mortality [5]. However, no medications are specifically approved to treat anorexia nervosa, and restoration to a healthy weight (or BMI), as the primary therapeutic goal, is generally pursued through psychotherapy and nutrition rehabilitation [6].

The ABA model provides an easy way to implement biobehavioral phenomena and is considered the most fitting animal model similar to AN. So far, many versions of ABA protocols have been in use, and consequently, several influencing factors have been described [7,8,9]. Our aim was to assess how accurately the standard acute ABA protocol mimics some of the most common peripheral AN complications—including metabolic, cardiovascular, and gonadal dysfunctions depending on gender, age, and initial body weight—to form a comprehensive description of ABA as a reliable research tool.

## 2. Materials and Methods

### 2.1. Animals

Wistar rats weighing 170–240 g (mean body weight: females = 195.2 ± 2 g, males = 200.2 ± 6.5 g; Jagiellonian University Medical College Animal Laboratory, Krakow, Poland) were included in the experiment. Upon arrival, the animals were housed for a week under controlled conditions—12 h light/12 h dark cycle and temperature of 22 ± 2 °C. Transparent cages were placed adjacent to each other to provide sight, acoustic, and odor contact. All cages contained suitable bedding materials and environmental enrichment. Animals were fed with standard dry chow: protein 25%, fat 8%, carbohydrates 67%, metabolizable energy 2.86 kcal/g (Labofeed B, Kcynia, Poland). Tap water was always available ad libitum. This study was carried out following ethical, regulatory, and scientific principles (protocol numbers 65/2017, 116/2018, 157/2018, 331/2019, and 445/2020).

### 2.2. Experimental Design

**Figure 1:**
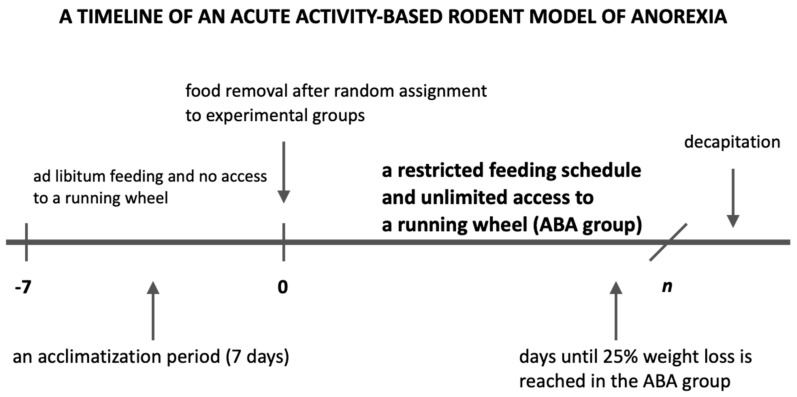
The general timeline of the ABA protocol used in the study. *n* = 6 for 19 ABA rats; *n* = 7 for 23 ABA rats (*n* = days until decapitation).

After an initial acclimatization period, with no access to a running wheel and ad libitum access to food and water, female (*n* = 88) as well as male rats (*n* = 20) were randomly assigned to one of four groups:

1. Control group (C group): animals placed in standard cages (without any access to a running wheel), with no extra physical activity and an ad libitum feeding schedule (*n* = 22);
2. Restricted Feeding group (RF group): animals placed in standard cages, with no extra physical activity and a restricted feeding schedule with access to food limited to 1.5 h per day at the end of the light phase (*n* = 23);
3. Wheel group (Wh group): animals placed in cages with unlimited access to a running wheel and an ad libitum feeding schedule (*n* = 21);
4. Activity-Based Anorexia group (ABA group): animals placed in cages with unlimited access to a running wheel and a restricted feeding schedule with access to food limited to 1.5 h per day at the end of the light phase (*n* = 42, Figure 1).

In order to ensure that food availability was time-restricted according to the experimental design, the cage bedding was refreshed immediately after the feeding, and the bottom of cages were additionally wiped to remove all remaining food crumbs and dust. The Wh and ABA rats were housed in cages with an activity wheel (Tecniplast 2154F0105 Activity Cage System for Rats).

### 2.3. Food Consumption and Physical Activity

The general health status of the experimental animals was evaluated daily during handling and by observing their in-cage behavior. At the same time, handling of animals was kept to a minimum since ABA is an example of a biobehavioral phenomenon, and it was critical to minimize the amount of unpredictable stress and maximize comfort for experimental animals. Body weight (g) and physical activity were monitored once a day before feeding between 4 p.m. and 6 p.m. The physical activity of an animal was determined based on the number of turns made on the wheel during 24 h. The experiment was discontinued and animals euthanized when the body weight loss exceeded 25% or humane endpoint criteria were met. Animals were first anaesthetized with an injection of ketamine/xylazine and then euthanized by decapitation.

### 2.4. Electrocardiography (ECG), Heart Rate Variability (HRV), and Systolic Blood Pressure (sBP) Measurements

ECG, HRV, and sBP were measured in randomly selected female rodents from ABA (*n* = 10), RF (*n* = 5), and Wh (*n* = 5) groups one day before the start of the experiment and on the last day of the experiment. It was performed in a selected group of animals to minimize the impact of an additional stress factor on the results of other measurements covering the entire study population. Before each measurement of heart rate variability, rats were anesthetized by intraperitoneal injection of ketamine/xylazine. Then, the chest and joints were gently shaved to facilitate electrode sticking, provide better signal quality, and narrow the incidence of artifacts. ECG and HRV measurements were carried out using LabChart 5.4.2 Pro (ADInstruments, Sydney, Australia). The animals were kept supine during anesthesia and testing, at a constant and appropriate body temperature provided by a heating pad with a rat temperature sensor (Physitemp, New York, NY, USA). RR interval data were collected from a 20 min ECG recording. A thorough visual inspection of the recording was performed to exclude artifacts. Each value of the ECG parameter was obtained from the mean of the five independent PQRST complexes. The parameters used in the analysis are presented in Table 1.

sBP was measured using a specialized tail cuff by periodic occlusion of tail blood flow (NIBP, AdInstrument, Sydney, Australia). Time-domain and frequency-domain heart rate variability parameters were obtained using Kubios software (Kubios Oy, Kuopio, Finland). We obtained the standard deviation of all NN intervals (SDNN, ms) and the root-mean-square of the successive differences (RMSSD, ms) to estimate sympathetic and parasympathetic activity in the time domain. For the frequency domain, the power spectrum was obtained using the autoregressive model. In addition, the analysis was supplemented with a set of nonlinear parameters.

### 2.5. Morphological Analysis of the Female Reproductive System

#### 2.5.1. Tissue Processing

Immediately after euthanasia, tissue fragments from rat uterine horns and ovaries were collected. Fresh tissue specimens were rinsed thoroughly with PBS (phosphate-buffered saline, 0.01 M, pH = 7.4), fixed with 9% phosphate-buffered paraformaldehyde, routinely processed, and embedded in paraffin (FFPE). Solidified paraffin blocks were cut into 5 μm thick sections and placed on slides with increased adhesion (Super Frost Plus).

#### 2.5.2. Hematoxylin–Eosin Section (HE) Staining

Hematoxylin–eosin (HE) staining for routine evaluation was performed on FFPE sections. The preparations were deparaffinized in xylene and then hydrated in alcohol at the concentrations of, respectively, 100%, 95%, and 70% for 5 min each. The preparations were then rinsed in distilled water for 5 min, incubated with Harris Hematoxylin for 10 min, and rinsed under running tap water for 20 min, then discolored with 1% acidic alcohol (1% HCl in 70% alcohol) for 5 min and washed under running tap water. The preparations were then immersed in an alkaline solution until reddening and washed with tap water. Next, the preparations were incubated with eosin for 2 min and rinsed in water for 1–5 min. In the last stage, the preparations were again dehydrated with alcohol (concentration from 70% to 100%), cleared in xylene three times for 1–2 min, dried, and closed in the DPX medium (Fluka).

#### 2.5.3. Immunofluorescence (IF)

After deparaffinization, rehydration, and antigen retrieval in citrate buffer, the slides were incubated for 30 min in PBS with appropriate normal serum at room temperature, which was followed by overnight incubation at 4 °C in a solution of PBS with appropriate normal serum containing a mixture of primary antibodies. After 5 washes (10 min each) in PBS, the specimens were then incubated for 1 h at room temperature with a mixture of secondary antibodies diluted in PBS. Indirect double immunofluorescence for identification of sex steroid hormone receptors in sections was performed with monoclonal mouse estrogen receptor (1:50; NCL-L-ER-6F11, Leica Biosystems), monoclonal mouse progesterone receptor (1:100; Clone PgR636, Dako), monoclonal mouse anti-Inhibin β-A antibody (1:100; sc-166503, Santa Cruz Biotechnology), and secondary goat anti-mouse Alexa 488-conjugated antibody (1:400; 115-545-146, Jackson ImmunoResearch). Appropriate negative and positive controls were performed. Finally, the slides were washed with two changes (10 min each) of PBS, cover-slipped with fluorescence mounting medium (Dako, Denmark), and covered with Menzel-Gläser glasses. The labeled specimens were analyzed immediately.

#### 2.5.4. Microscopy

Microscopic preparations after HE staining were analyzed using an Axiophot (Zeiss) light microscope. IF images were obtained using an MN800FL epifluorescence microscope (Opta Tech, Warsaw, Poland). Photographic documentation was taken with an Olympus DP74 camera coupled to the microscope. Digital images were collected at 40×, 100×, 200×, or 400× magnifications. The qualitative analysis of cells was performed in 10 consecutive high-power fields (200×) using the computer-based image analysis system software Multiscan 18.03 (CSS, Warsaw, Poland). All samples were assessed by two independent specialists (each blinded to the other) without any knowledge of the clinical parameters or other prognostic factors to avoid bias. In all sections, the estrogen and progesterone receptor expression and the Inhibin B expression were evaluated with respect to the relative frequency (arbitrarily graded as no expression = 0, low expression = +, moderate expression = ++, high expression = +++).

### 2.6. Biochemical Analysis

Blood samples from the jugular vessels were collected in plastic tubes immediately after decapitation and incubated at least 30 min at 4 °C to induce clot formation. After centrifugation at 1500× *g* for 20 min at 4 °C (Megafuge 1.0R, Heraeus Instruments, Germany), serum samples were separated and kept frozen in small volumes at −20 °C until further analysis. Samples were thawed immediately before the assays. All measurements were performed in duplicate. Luteinizing hormone (LH) and follicle-stimulating hormone (FSH) levels were determined in the blood serum using immunoenzymatic methods, according to the manufacturer’s (DRG Instruments GmbH, Germany) instructions. Glucose (GLC), aspartate aminotransferase (AST), alanine aminotransferase (ALT), total cholesterol (CHOL), HDL, LDL, and triglycerides (TG) levels were determined in the blood serum using photometric assays measured with Roche/Hitachi Cobas c 701 and Roche/Hitachi Cobas c 501 analyzers.

### 2.7. Assessment of Adipose Tissue Volume by Magnetic Resonance Imaging (MRI)

MRI was used to determine the volume of visceral and subcutaneous adipose tissue at the level of the L4 vertebra in healthy and ABA rats. MRI experiments were performed on a 9.4T Bruker Biospec scanner, using a Bruker T9361 transmit/receive birdcage coil with a diameter of 72 mm. Animals were euthanized with pentobarbital prior to imaging (Morbital, Biowet, Pulawy; 1–2 mL/kg b.wt.) after ketamine/xylazine premedication to obtain high-quality images devoid of any artifacts. Imaging was performed using the fast spin-echo rapid acquisition with a relaxation enhancement sequence (RARE). Pulse sequence parameters were echo time = 6.3 ms, repetition time = 2000 ms, RARE factor = 8, acquisition matrix = 256 × 256, field of view = 60 × 60 mm, and number of slices = 23. The entire abdomen from L1 to L6 was covered with 1 mm thick axial slices. Imaging was done twice, first without fat suppression (RARE) and repeated with the fat suppression module (fsupRARE) turned on. In the images obtained with the spin-echo sequence, fat appears as a bright area. This area becomes correspondingly darker when fat suppression is used. This relationship was used to find image pixels containing fat. Adipose tissue segmentation was performed using a custom-developed MATLAB script (The Mathworks, Inc.). The slice passing through the L4 vertebra in the reference anatomical image was selected for further analysis. First, the pixel values of both images were normalized to 1. The difference between the normalized RARE and fsupRARE images was then calculated—the fat containing pixels were those for which the difference image pixel value was greater than 0.2. Finally, adipose tissue volume was calculated as the product of the number of pixels containing fat and the volume of a single pixel.

### 2.8. Statistical Analysis of the Results

Numerical results are presented in mean form with standard error (SEM) or standard deviation (SD). Most of the data were analyzed using one-way analysis of variance (ANOVA) followed by a Games–Howell or Tukey post hoc test. Outliers were assessed by inspection of a boxplot for values greater than 1.5 box-lengths from the edge of the box. The two-tailed paired Student’s *t*-test was used to compare differences between two dependent groups. In some cases, to meet the assumption of normality, the data were transformed using the logarithmic function (log 10). Correlations were tested using Pearson’s correlation coefficient. The differences when *p* < 0.05 were considered statistically significant. The calculations were performed using IBM SPSS Statistics for Mac, Version 27.0. Armonk, NY: IBM Corp (licensed to Jagiellonian University).

## 3. Results

### 3.1. Reduction of Body Weight

The control males gained weight more rapidly, reaching almost 114% of their initial weight compared with females that grew to 106% (13.93 ± 0.57 vs. 6.01 ± 0.82; *p* < 0.05).

A one-way Welch ANOVA was conducted to determine the difference in the final weight loss between the groups. There were six outliers in the data. Those outliers were removed from the analysis. The activity-based anorexia model induced the most substantial weight loss compared with the Wh (mean difference = 22.2 ± 1.1%, *p* < 0.001) and RF (mean difference = 7.7 ± 0.7%, *p* < 0.001) groups. At the beginning of the experiment, RF caused body weight loss to the same extent as ABA. Mean daily weight loss in RF and ABA was statistically equal during first 2 days in females (D1: 6.0 ± 0.3 vs. 7.7 ± 0.7, *p* = 0.161; D2: 8.9 ± 0.4 vs. 10.4 ± 0.5, *p* = 0.126) and 3 days in males (D1: 9.1 ± 0.3 vs. 7.6 ± 0.5, *p* = 0.13; D2: 11.9 ± 0.5 vs. 12.3 ± 0.6, *p* = 0.955; D3: 13.6 ± 0.5 vs. 15.4 ± 0.6, *p* = 0.141).

**Figure 2:**
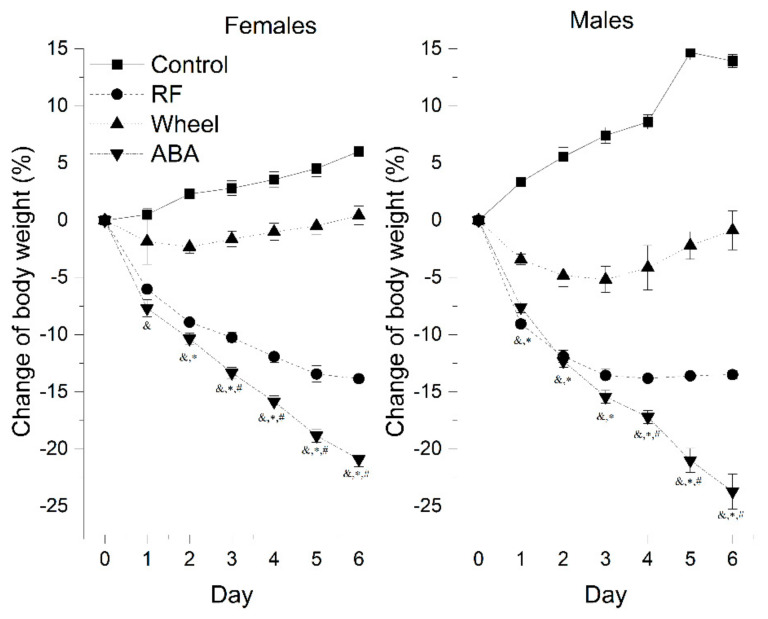
Body weight change in the all experimental groups of females (*n* = 82) and males (*n* = 20). Statistically significant difference marked as: ^&^ vs. C, ^#^ vs. RF, * vs. Wh.

In subsequent days, the ABA weight loss accelerated drastically. In each other group, the values differed significantly on individual days, presenting a different rate of body weight change depending on the experimental factors (Figure 2). Running wheel exposure caused an initial decline of body weight, but its anorectic effect alleviated during the experiment. Both females and males independently showed similar patterns of weight loss, i.e., there were no statistically significant differences between sexes in the experimental groups based on the final effect of each model on body mass.

Detailed comparison between sexes found that:

1. There were no significant differences in the development of the ABA weight loss pattern.
2. In the Wh group, males lost weight more rapidly than females on the second and third day. They presented an upward trend one day longer than females, followed by a gradual return to the initial weight.
3. In the RF group, males presented a significantly more intensive weight loss up to the fourth day, to stabilize after reaching a peak at levels comparable with females that presented a stable, gradually increasing pattern.

When we segregated female animals based on the 200 g weight criterion (>200 g: *n* = 37, mean initial body weight (iBW) = 216.9 ± 1.9 g; <200 g: *n* = 45, iBW = 180.6 ± 1.6 g), we observed that heavier animals were less prone to wheel exposure alone, as the difference compared with controls was not statistically significant at any point of the experiment. However, controls >200 g gained 2.2 ± 1.5%, while those <200 g gained 7.3 ± 0.7%, and the final weight reductions in both weight-determined Wh groups were comparable (Wh >200 g: 0.2 ± 1% vs. Wh <200 g -1.9 ± 1.2%). On the other hand, food restriction in larger rats led to virtually the same effect as ABA up to day 5 (final weight difference = 5.6 ± 1.9%; *p* = 0.039). In rats weighing less than 200 g, the statistically significant difference between RF and ABA developed earlier, starting on day 4, and attained greater values (final weight difference = 6.3 ± 0.8%, *p* < 0.001).

### 3.2. Physical Activity

The activity of rats exposed to voluntary activity in a running wheel (ABA vs. Wh group) systematically grew as the experiment progressed. ABA rats showed remarkably increased activity compared with Wh and reached the statistical significance observed from the third day. At the end of the experiment, ABA exhibited almost 177% more intense activity, based on the number of daily revolutions of the wheel, than the animals in the wheel group (ABA: 32,402 ± 2368 vs. Wh: 11,706 ± 2457; *p* < 0.001).

**Figure 3:**
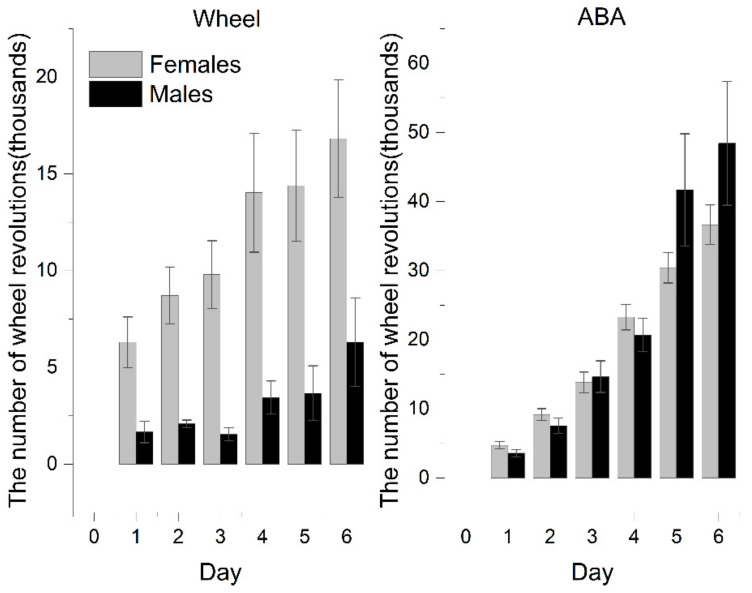
Physical activity of female and male rats in the wheel (Wh; *n* = 21) group and activity-based anorexia (ABA; *n* = 42) group.

Females were more susceptible to wheel activity exposure (Figure 3). In the wheel group, females exhibited significantly increased physical activity than males. It results in a more pronounced difference between male ABA and Wh (on the last day of the experiment: ABA: 48,415 ± 8957 vs. Wh: 6306 ± 2280; *p* < 0.05).

Female rats with iBW exceeding 200 g showed an activity pattern that was the same as that of the whole experimental population, with a clear distinction between ABA and Wh. However, this difference was less pronounced in those who were included in the experiment with weights less than 200 g as a result of the increased activity of the Wh rats (on the last day of the experiment: ABA: 38,521 ± 3391 vs. Wh: 24,625 ± 4409; *p* = 0.066).

### 3.3. Food Consumption

The time-limited feeding schedule crucially reduced the absolute amount of consumed food. The RF and ABA groups ate significantly less compared with the Wh and control. However, the accessibility of the running wheels also negatively affected food consumption in those subsets of animals included in the similar feeding schedule. A one-way Welch ANOVA was conducted to determine if the average food consumption was different for the experimental groups regardless of weight and sex. There were ten outliers in the data that were removed from the analysis. Data were normally distributed for each group, as assessed by the Shapiro–Wilk test (*p* > 0.05), but there was heterogeneity of variances, as assessed by Levene’s test of homogeneity of variances (*p* < 0.001). The average food consumption was statistically significantly different between the groups (Welch’s F(3, 31.862) = 79.172, *p* < 0.001). The average food consumption decreased from the control group (16.08 ± 0.8) to the Wh (10.1 ± 1.2), RF (5.4 ± 0.2), and ABA (4.2 ± 0.2) group, in that order. Games–Howell post hoc analysis revealed that the difference was statistically significant between C and Wh (5.9 ± 1.4, *p* = 0.001), as well as RF and ABA (1.2 ± 0.3, *p* < 0.001).

**Figure 4:**
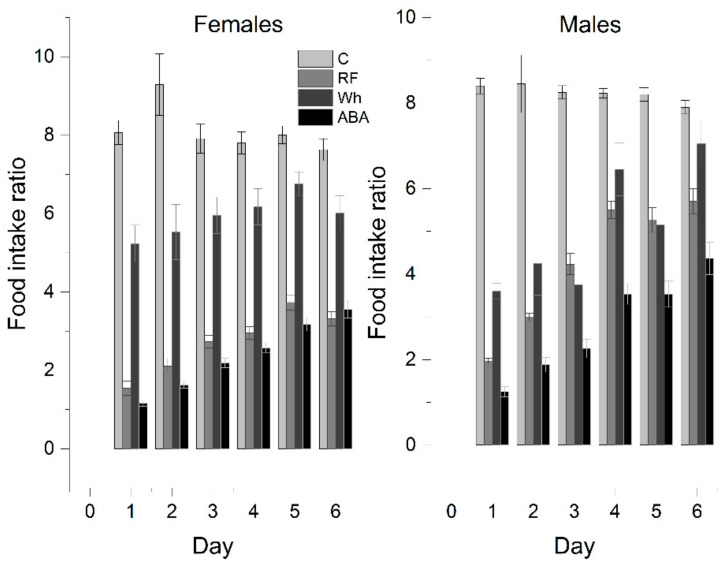
Food consumption of the experimental rats calculated as grams of food per 100 g of body weight (food intake ratio).

In further analysis, to exclude body weight bias, the food intake rate (IR) was recalculated as the weight of the pellets consumed per 100 g of body weight adjusted each day (Figure 4). It allowed to conduct a one-way ANOVA with Tukey post hoc analysis that confirmed the average IR was significantly higher in the control group than in any other group, and the differences were significant between all experimental groups (total daily average—C: 8.1 ± 0.2, RF: 3.1 ± 0.2, Wh: 5.7 ± 0.2, ABA: 2.5 ± 0.1). A one-way repeated measures ANOVA was performed in the ABA group to determine if there were statistically significant differences in food intake over the course of the 6-day experiment. The assumption of sphericity was violated, as assessed by Mauchly’s test of sphericity. Therefore, a Greenhouse–Geisser correction was applied (ε = 0.554). The average food intake increased over time (*p* < 0.001). Post hoc analysis with a Bonferroni adjustment revealed that food consumption increased statistically by day 5 (difference between days 5 and 6 = 0.42 ± 0.1, *p* = 0.098).

Females:

Controls and Wh ate similar absolute amounts, but the average Wh food intake rate was almost 30% lower than the control (C: 8.1 ± 0.3 g vs. Wh: 5.9 ± 0.2 g; *p* < 0.05), even though the Wh rats showed a negative change in body weight for most of the experiment—Wh did not compensate for the increased physical activity with adequate consumption. IR for RF (2.7 ± 0.1 g) and ABA (2.4 ± 0.1) were comparable (*p* = 0.143); therefore, increased voluntary activity between feeding periods appears to be the major factor in escalating weight loss.

Males:

Similarly, IR was the highest for controls (C: 8.3 ± 0.2 vs. Wh: 5.0 ± 0.4; *p* < 0.05). However, wheel exposure reduced food intake to the same extent as restricted feeding (RF: 4.3 ± 0.1; *p* = 0.374) due to relatively higher consumption in the male than the female RF group. Both factors combined resulted in the lowest IR (ABA: 2.9 ± 0.5).

The difference between RF and ABA gained significance in animals with iBW below 200 g (RF: 3.6 ± 0.2, ABA: 2.5 ± 0.1; *p* < 0.05). An iBW above 200 g determined similar consumption in both RF (2.6 ± 0.2) and ABA (2.2 ± 0.1; *p* = 0.339). The difference between the C and Wh groups was less noticeable (no significant difference on D3, D4, and D6), mostly due to smaller IR in heavier animals (Controls: <200 g: 8.7 ± 0.3, >200 g: 7.5 ± 0.2). Moreover, there was a significant correlation between iBW and IR in the RF group (–0.804, *p* < 0.01; 2-tailed). No such correlation was observed in Wh or ABA.

### 3.4. Biochemical Parameters

Glucose:

Rats upon exposure to the ABA paradigm developed severe hypoglycemia compared with other groups in which glucose levels were fairly the same (Table 2). Glycemia was statistically significantly higher in non-ABA groups than in the ABA group (a mean difference of 4.1 ± 0.4, *p* < 0.001).

Aminotransferases:

The liver enzymes were strongly affected by increased physical activity, with a dramatic elevation in their levels. Both ALT and AST were significantly higher (a mean difference of 18.3 ± 4 for ALT and 62 ± 13 for AST, *p* < 0.001) in the running groups (ABA and Wh) compared with the non-running groups (C and RF). Wh showed a similar trend as ABA, although AST in the Wh group did not reach a significance threshold when compared with controls. Food restriction did not affect the concentration of transaminases.

Lipid profile:

The ABA rats showed a cumulative decline with extremely low levels of all parameters compared with the other groups (*p* < 0.05). Fluctuations of lipid profile in the Wh group may seem to imitate less intense ABA. However, triglyceride levels were the only ones influenced by Wh to the same extent as by ABA (TG: Wh vs. ABA, *p* = 0.714). For total cholesterol and HDL, Wh showed a significant reduction compared with controls, but LDL levels were elevated (CHOL, HDL: Wh vs. C, *p* < 0.05; LDL: Wh vs. RF, *p* < 0.05). Food restriction markedly affected only triglyceride concentrations (TG: RF vs. C, *p* < 0.05).

### 3.5. Body Composition

**Figure 5:**
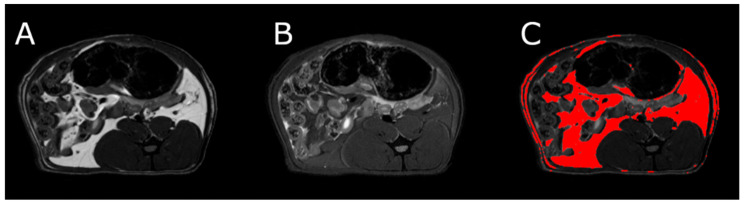
MRI scan of female healthy rat, the cross section of the abdomen at L4 level. (**A**) RARE image without fat suppression; (**B**) fsupRARE image with fat suppression; (**C**) segmentation result—adipose tissue marked in red.

**Figure 6:**
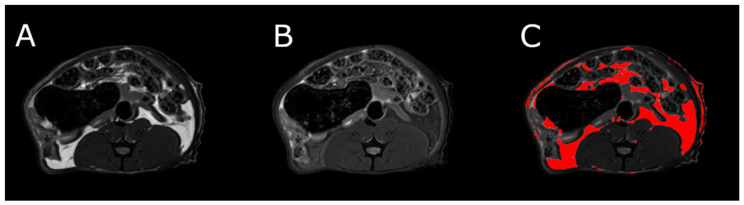
MRI scan of female ABA rat, the cross section of the abdomen at L4 level. (**A**) RARE image without fat suppression; (**B**) fsupRARE image with fat suppression; (**C**) segmentation result—adipose tissue marked in red.

MRI measurements showed a significant reduction in the adipose tissue volume of ABA rats compared with controls (Figure 5). The mean visceral body fat reduction in the ABA rats reached 58.9% on day 6 of the experiment (Figure 6).

### 3.6. Female Reproductive System

Uterine weight was 2 times lower in a rat model of an activity-based anorexia nervosa when compared with the control group (Table 3).

A detailed microscopic analysis of the morphological structure of the uterine horn was performed in ABA and C groups, including the endometrium (surface epithelium and lamina propria) and the myometrium layer. There was no difference in wall thickness, while the endometrium was thinner in the ABA group.

**Figure 7:**
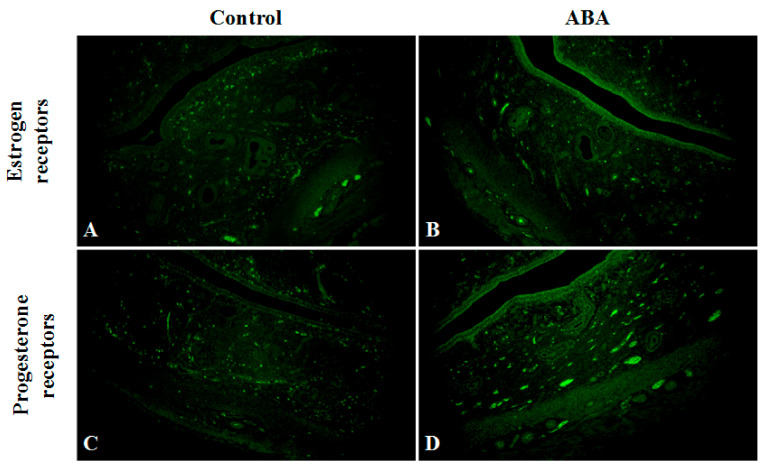
Uterine horn in the control group (**A**,**C**) and ABA (**B**,**D**) group stained for estrogen (green, Alexa Fluor 488) and progesterone (green, Alexa Fluor 488) receptors. Expression of both receptors in the endometrium of ABA rats is reduced. Total magnification: ×200.

The expression of estrogen receptors in the ABA group decreased. The receptors detected, in most cases, were located near the myometrium. However, the expression of progesterone receptors was slightly lower in the ABA group compared with the control group (Figure 7).

This morphological presentation corresponds with the level of sex hormones in blood samples from each group. FSH levels were 4.5 times higher in ABA rats, with no difference in LH levels between the two groups.

**Figure 8:**
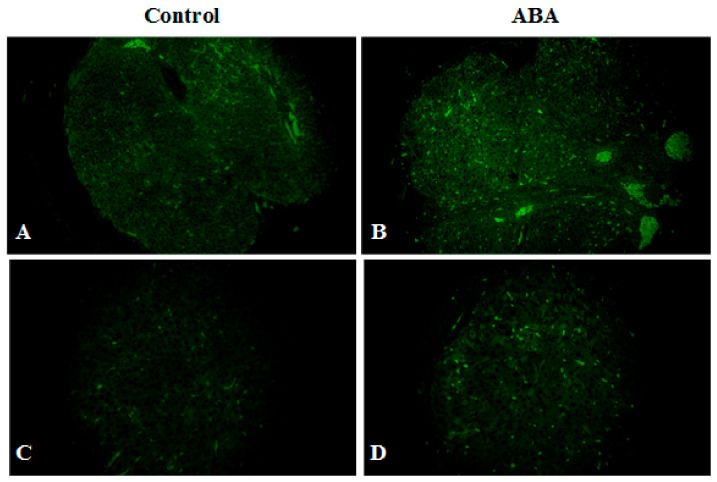
Ovarian tissue from the control group (**A**,**C**) and ABA (**B**,**D**) group stained for Inhibin B (green, Alexa Fluor 488). Preantral follicles are positive for the current marker, while pre-dominant and dominant follicles are negative for Inhibin B. Total magnification: ×200 (**A**,**C**); ×400 (**B**,**D**).

**Figure 9:**
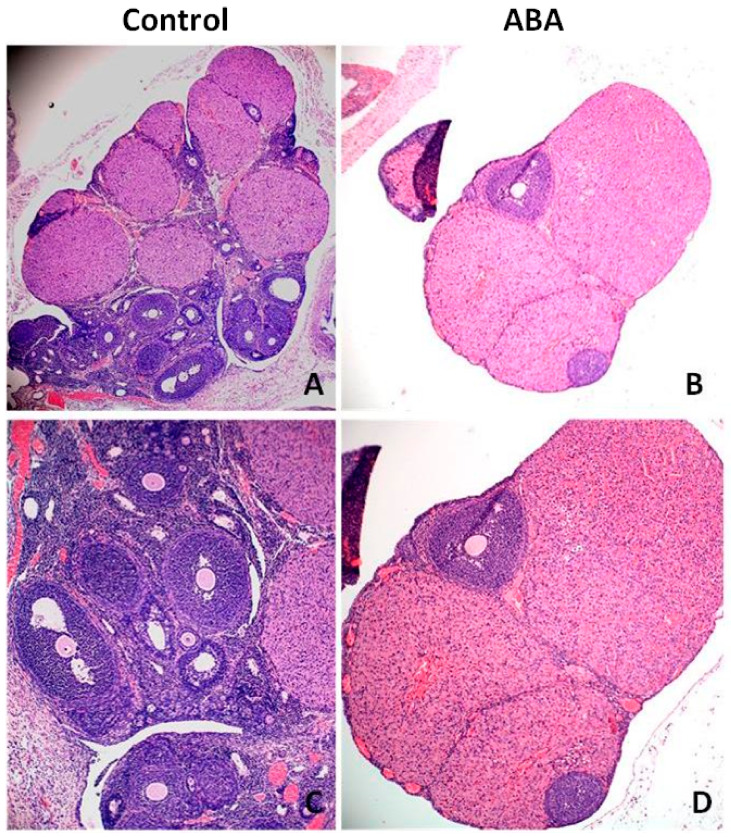
Hematoxylin–eosin-stained cross sections of rat’s ovaries with antral follicles in the control group (**A**,**C**) and ABA group (**B**,**D**). The ABA group is characterized by a poor follicle pool; only a few follicles were observed in the marginal part of ovaries. Total magnification: 40× (**A**,**B**); 100× (**C**,**D**).

Microscopic analysis of ovarian tissue revealed a reduction in the number of antral follicles (Figure 8). The ovaries of healthy rats contained more follicles at various stages of growth, including the dominant follicle, while the ovaries from the ABA group had a poor pool of follicles without a leading follicle. The expression of Inhibin B was significantly higher in the ABA group (Figure 9).

### 3.7. Electrocardiography

**Figure 10:**
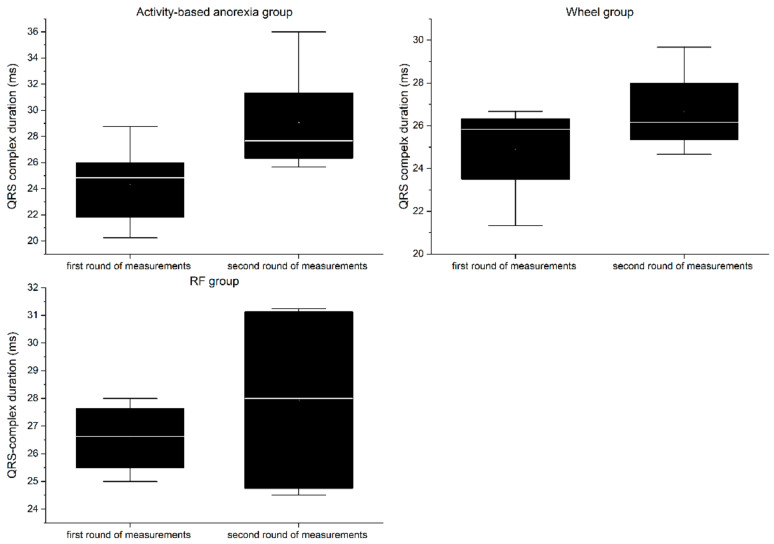
Boxplot of mean QRS-complex duration in the experimental groups. The first round of measurements serves as control.

**Figure 11:**
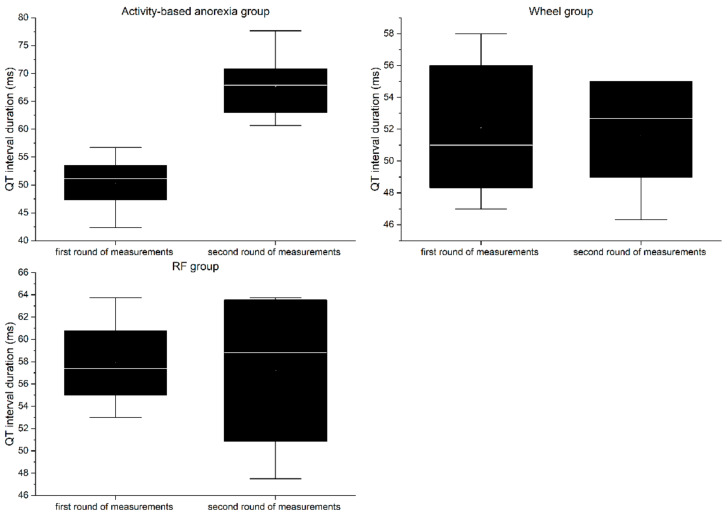
Boxplot of mean QT-interval duration in the experimental groups. The first round of measurements serves as control.

RP group:

In the first round of ECG recordings, the QRS complex (Figure 10) and QT interval (Figure 11) lasted on average 26.56 ± 1.33 ms and 57.88 ± 4.44 ms, respectively. In the second round, the duration was 27.94 ± 3.69 ms for the QRS complex and 57.2 ± 7.82 ms for QT interval. The differences between the two consecutive measurements were not statistically significant for any of the parameters (QRS = 1.38 ms, QT = 0.68 ms; *p* > 0.05).

Wheel group:

In the first round of ECG recordings, the mean duration of the QRS complex and QT interval was 24.92 ± 2.42 ms and 52.07 ± 4.78 ms, respectively. In the second round, the duration was 26.67 ± 2.13 ms for the QRS complex and 51.6 ± 3.83 ms for the QT interval. The differences between the two consecutive measurements were not statistically significant for any of the parameters (QRS = 1.38 ms, QT = 0.47 ms; *p* > 0.05).

ABA group:

In the first round of ECG recordings, the average QRS complex and QT interval lasted 24.29 ± 1.01 ms and 50.39 ± 4.63 ms, respectively. In the second round, the duration was 29.04 ± 3.62 ms for the QRS complex and 67.73 ± 5.6 ms for the QT interval. ABA modeling led to a significant prolongation of both the QRS complex (4.75 ms, *p* < 0.05) and QT interval (17.34 ms, *p* < 0.01).

### 3.8. Heart Rate Variability

RF group:

Although there was no statistically significant impact of food restriction on HRV parameters (Table 4), there was a visible shift in the measured values towards parasympathetic dominance. Both LF power (%) and LF power (n.u.) were lowered, representing decreased sympathetic activity (Table 5). The lowered LF/HF ratio mirrored these findings. Lowered nonlinear indexes such as SD2 and DFA1 were also associated with decreased sympathetic activity (Table 6). ApEn and SampEn values were similar to those in the control group.

Wh group:

In this group, the highest values of HR min and HR max were found. The sympathovagal balance was comparable with the RF group. ApEn and SampEn measurements gave results similar to the control and RF groups.

ABA group:

Despite the highest values of mean RR (statistically not significant, *p* > 0.05) and SDNN (statistically significant, *p* < 0.05), acquired data presented sympathetic dominance in anorectic rats (LF (n.u.), LF/HF ratio, SD2, SD2/SD1, and DFA1). Entropy was significantly reduced compared with the other groups (*p* < 0.05). Moreover, despite the decrease in heart rate, there was an increase in systolic blood pressure (*p* > 0.05).

## 4. Discussion

Eating disorders, including anorexia nervosa, can affect people of all genders, ages, races, sexual orientations, body shapes, and weights. What is more, the on-going pandemic has substantially affected individuals with eating disorders [10]. AN has the highest mortality rate (12 times higher than of all causes of death for females aged 15–24 years) of any psychiatric disorders [5]. Nonetheless, the increasing prevalence of AN does not run parallel with the available treatment options. The importance of psychological and socio-cultural factors in the development of AN is widely established; however, metabolic dysregulation is evident as well [11]. Thus, it would be unrealistic to build an animal model upon the entire AN pathology. Still, ABA is not only highly similar to AN, but it is also considered the best animal model of any psychiatric disorder [12,13,14]. ABA successfully captures numerous clinical features of anorexia nervosa, such as severe weight loss, hunger-induced hyperactivity, anxiety, cessation of the estrous cycle (females), and heightened vulnerability during puberty [7]. ABA has been performed in the same manner in various rodent breeds or species, including rats (in the majority of cases either Sprague-Dawley or Wistar). In general, due to the spontaneous variability in activity, about 20–30% of rats should not be interested in running [15], which we did not observe in the first instance. What is more, rodent males and females exhibit different patterns of physical activity after limitation of food access [16]. It was already reported that wheel running should be significantly greater and weight loss should occur more quickly in female rats than in male rats [1,17,18], which have been related to higher rates of wheel running in female rats. Sex- and age-dependent increase of voluntary activity may have its origin in the evolutionary concept that females are more active due to the necessity of obtaining more food to meet the needs of their offspring. Its biological basis may depend in part on sex hormones (higher estrogen levels and lower testosterone levels than in males), which influence specific brain regions, such as the striatum or nucleus accumbens, via pathways related to dopamine signaling [19]. Our data confirmed that female gender and lower weight favors more intense physical activity, but in ABA animals, the influence of these determinants is less evident. We observed that both females and males showed similar patterns of weight loss in the ABA model. Regardless of their initial weight, ABA rats ate the same relative amount of food. However, in smaller animals, running wheel exposure caused an initial “exaltation phase”—rodents with constant access to the activity wheel voluntarily reduced their food intake despite increased energy requirements and unrestricted access to food. In combination with time-limited feeding, it provoked even more intensive running and thus greater weight loss in animals with a lower body weight. Larger animals were significantly less interested in voluntary physical activity, while feeding restriction in this population escalated to almost the same extent as in ABA rats. Males in particular were more prone than females to more aggravated weight loss in the face of a food-limited schedule. It is worth noting that males represent about 25% of individuals with AN, yet they are at much higher risk of dying, partly due to initial misdiagnosis and inadequate interventions [20,21].

The negative effect of ABA can be monitored by commonly used biochemical parameters. As metabolism rearrangements due to caloric restriction and electrolyte abnormalities can lead to liver cell damage, abnormally high levels of transaminases can be observed in patients with anorexia as well as in ABA rats. Serum ALT and AST concentrations correlate with the severity of anorexia nervosa [22]. Their increase is directly related to lower BMI, lower body temperature, and lower pulse rate. Nevertheless, transaminases are present in many other tissues, such as the heart, kidneys, and muscle, so damage to these tissues can also lead to an increase in transaminase levels. Elevated concentrations of total cholesterol (TC), LDL, and HDL are also observed, being potentially the effect of accelerated cholesterol metabolism [23]. These alterations are accompanied by commonly detected hypoglycemia. As with severe anorexia, ABA rats showed elevated transaminases due to liver dysfunction. Similarly, as the glycogen stores in the liver are consumed and the process of gluconeogenesis becomes ineffective, glucose levels of ABA rats decrease. It is suspected that hepatocyte damage and subsequent death are the result of ischemia, glutathione deficiency, or starvation-induced cellular autophagy [24,25,26]. The latter, regulated by the concentration of amino acids, glucagon, and insulin, leads to the degradation of cell content, which can be particularly harmful in the case of severe malnutrition. Moreover, strenuous physical activity has been implicated as a potential cause of hepatic enzyme elevation, which is further supported by the observed increase in AST and ALT in the Wh group [27]. On the other hand, contrary to the lipid profile of patients with AN, we observed a reduction in the concentration of all four parameters (TC, TG, LDL, and HDL) in the ABA animals. Such a decrease in lipid and lipoprotein levels resembles a critical state of malnutrition, while in AN, increasing evidence points to more complex dysregulation of lipid metabolism [28].

Female rodents subjected to ABA, in addition to excessive activity, reduced food intake, and weight loss, present with the cessation of the estrous cycle. Reduction in the number of ovarian follicles and thickening of the endometrium in ABA rats seems to be the result of estrogen deficiency. We observed a compensatory increase in the serum concentration of FSH, which in turn stimulates the production of Inhibin B, one of the most important factors in follicle development. Inhibin B levels have been suggested to inversely correlate with body mass index, especially in patients with polycystic ovary syndrome. In AN women, the ovulation block results from hypogonadotropic hypogonadism, defined as dramatically low levels of luteinizing and follicle-stimulating hormones. Underlying hypothalamo-pituitary-gonadal axis dysfunction, with improper secretion of the key regulator of this process, gonadotropin-releasing hormone (GnRH), is caused by a complex neuroendocrine mechanism that involves interactions of numerous hormones such as leptin, ghrelin, adiponectin, or peptide YY [29]. There is a direct correlation between the resumption of menstruation and the increase in body weight, ovarian volume, and uterine volume [30]. It should be mentioned that although amenorrhea as a diagnostic criterion was removed from the *Diagnostic and Statistical Manual of Mental Disorders, Fifth Edition* (DSM-5) [31], only a minority of females with anorexia nervosa continue menstruating despite extreme weight loss and malnutrition, and thus the resumption of menses is an important indicator of recovery from AN [32].

The results of electrocardiographic measurements show that animals included in the ABA paradigm developed severe impairment of the functioning of the cardiac conduction system. Prolongation of both the QRS complex and the QT interval indicates disturbances in intraventricular impulse conduction and cardiac repolarization. Similar changes were reported in patients suffering from AN [4,33,34,35]; however, some studies have not revealed significant changes in the QT-interval [36,37], making this parameter a questionable diagnostic tool [38]. Nevertheless, QT-interval prolongation is associated with increased mortality. Proposed pathologies underlying the described conduction complications include cellular changes in myocardiocytes secondary to malnutrition [34], hypokalemia, or anatomical remodeling of the heart [39]. Although we have not been able to identify the exact pathophysiological background of the observed changes, the ABA model quite accurately mimics the basic electrical processes in an anorexic diseased heart.

Although Lechin et al. described increased adrenal sympathetic activity in patients with AN, they typically present with bradycardia, hypotension, increased HRV, and domination of parasympathetic modulation (increased SDNN and HF) [40,41]. In ABA rats, we observed a relatively increased sympathovagal ratio, but such interpretation based solely on several variables needs to be made cautiously. There was a visible shift in frequency-domain nominal values towards sympathetic dominance, but it was not statistically significant. Sympathetic influence was most strongly represented by nonlinear indices with reduced entropy estimates. These changes appear to reflect the disease decompensation phase, in line with reported physical and behavioral symptoms at the end of ABA modeling. In its initial stage, the increase in parasympathetic activity should be associated with adaptation to a low energy supply. This adaptation can break down over time, with an increase in sympathetic/parasympathetic balance that is associated with a greater risk of cardiac death. Thus, the activity of the autonomous system may depend on the disease severity. Several factors that may have blurred the observed impact of ABA on HRV, including ketamine/xylazine anesthesia, which has been reported to increase parasympathetic and suppress sympathetic activity [42]. Moreover, there is a lack of direct evidence of hypokalemia, heart remodeling, or myocardiocyte changes, which have been proposed to be responsible for conduction changes among human patients. Recently, it has also been suggested that research into cardiovascular complications of AN may, along with the assessment of HRV, additionally benefit from the assessment of microvascular damage [43,44].

Anorexia nervosa is characterized by a very wide range of alterations of both central and peripheral origin. Dysfunction in the neural circuits of the reward system and/or appetite regulating neuropeptide pathways underlie some of the behaviors characteristic of patients with eating disorders [45,46]. AN patients usually present with anhedonia, anxiety, food aversion, and excessive physical activity, indicating abnormal reward processing [47]. And those patients do not adequately respond to homeostatic body signals such as appetite regulating peptides that should trigger weight restoration [46]. The central aspects related to AN etiopathogenesis (for a review see, Skowron et al. 2020) [11] are beyond the scope of this article; however, it should be noted that research using animal models, including the ABA model, has contributed immensely to the understanding of potential brain mechanisms that many underlie the causes and consequences of aberrant eating behaviors [48,49,50].

Although ABA is often compared with a simple starvation experiment, the model is an uncontrolled compounding of self-destructive reactions to the experimental environment. Weight loss seems to be initially an effect of restricted feeding, but despite the state of negative energy balance, it is subsequently potentiated by increased activity. With its rapid development, ABA successfully mimics the acute clinical features of AN; nevertheless, it is noteworthy that the model may not be adequate to study long-term AN complications such as bone mineral loss [51]. Moreover, direct translation of data obtained from ABA may be limited only to patients with hyperactivity (up to 80% of AN patients) [52]. It also appears that the reliability of ABA research interventions requires a unified methodology, as it has been shown that the basic features of the incorporated animals strongly influence the response to modeling. Adolescent female rats weighing less than 200 g are the preferable choice because of their increased susceptibility to ABA development, a more pronounced two-factorial presentation compared with food-restricted rodents due to inherent hyperactivity, which is a phenotype resembling a major part of the human AN population.

## 5. Conclusions

Even a general superficial look at the activity-based anorexia model reveals a fascinating phenomenon when animals voluntarily make a self-destructive choice between food consumption and physical activity. While its origins are undeniably much less complex, such behavior resembles that observed in patients with anorexia nervosa and leads to a series of common biological complications. Along with the relative ease of its implementation, these factors motivate scientists to choose this model frequently in their work on the pathophysiology of the disease. We made an attempt to analyze the basic features of the model in relation to the symptoms presented by patients with AN, which constitute a starting point for the investigated interventions. The analysis showed that rapid weight loss with increased physical activity produced a number of clinical symptoms of anorexia, including acute liver injury, hypoglycemia, lipid depletion, disruption of folliculogenesis, prolongation of the QT interval, and autonomic decompensation. Female and younger/smaller rats are more susceptible to wheel exposure and thus enhance the combined factors of ABA in favor of simple starvation-induced implications. We believe that the ABA model, with an awareness of its drawbacks, is a reliable research tool in animal studies on the course of ongoing anorexia involving behavioral and biological symptomatology.

## Figures and Tables

**Table 1:** Electrocardiography (ECG) parameters.

ECG Parameter	Definition
PR Interval	Time from the beginning of the P wave until the beginning of the QRS or RS complex
QRS complex	Time between the onset of R and the end of S
QT interval	Time from the onset of R complex to the end of T wave

**Table 2:** Biochemical analysis of the blood samples collected from 49 rats (both females and males). Data expressed as mean ± SEM. Statistically significant difference marked as: &—vs. C, #—vs. RF, *—vs. Wh, ^—vs. ABA (a one-way Welch ANOVA).

Parameter	Control	RF	Wh	ABA
GLC (mmol/L)	8.34 ± 0.14 ^	8.55 ± 0.54 ^	7.92 ± 0.4 ^	4.09 ± 0.38 &#*
AST (U/L)	112.2 ± 8.75 ^	104.08 ± 7.01 *^	155.8 ± 19.45 #	184.44 ± 12.88 &#
ALT (U/L)	34 ± 1.14 *^	30.2 ± 1.6 *^	51.3 ± 4.96 &#	54.92 ± 7.85 &#
CHOL (mmol/L)	1.52 ± 0.05 ^*	1.25 ± 0.1 ^	1.02 ± 0.06 &^	0.56 ± 0.07 &#*
HDL (mmol/L)	1.03 ± 0.04 ^*	0.81 ± 0.06 ^	0.67 ± 0.03 &^	0.23 ± 0.04 &#*
LDL (mmol/L)	0.2 ± 0.02 ^	0.23 ± 0.03 *^	0.47 ± 0.09 #^	0.13 ± 0.01 &#*
TG (mmol/L)	1.53 ± 0.08 #*^	0.57 ± 0.04 &^	0.42 ± 0.01 &	0.29 ± 0.03 &#

**Table 3:** Reproductive parameters and hormonal profiles in the control group and in animal model of anorexia nervosa. + = low expression, ++ = moderate expression, +++ = high expression.

	Control	ABA
Uterus		
Uterine weight (g)	1.085 ± 0.035	0.572 ± 0.153
Uterine horn: the wall thickness (μm)	1080 ± 141.7	1026 ± 116.3
Uterine horn: endometrium thickness (μm)	741.7 ± 150.9	574.6 ± 88.9
Expression of estrogen receptors	+++	+
Expression of progesterone receptors	+++	++
Reproductive hormones		
FSH blood level (mIU/mL)	8.18	35.9 ± 40
LH blood level (mIU/mL)	4.8 ± 4.0	5.0 ± 3.5
Ovary		
Number of follicles	16	2
The diameter of follicles (μm)	63 ± 219	636 ± 21
Inhibin B expression	+	+++

**Table 4:** HRV time-domain measures. Data expressed as mean ± SD. Statistically significant difference marked as: &—vs. C, #—vs. RF, *—vs. Wh, ^—vs. ABA.

	Mean RR (ms)	SDNN (ms)	RMSSD	Min HR (beats/min)	Max HR (beats/min)	pNN10
C	238.33 ± 18.36	3.85 ± 1.84 ^	4.61 ± 5.39	246.27 ± 18.89 *	264.27 ± 24.14 *	4.02 ± 7.95 ^
RF	228.6 ± 14.9	3.06 ± 1.11 ^	4.44 ± 2.22	257.4 ± 16.6	273 ± 17.9	5.27 ± 8.73
Wh	218.8 ± 18	3.08 ± 1.20 ^	3.28 ± 1.18	269.4 ± 24.2 &^	291.2 ± 23.2 &	0.02 ± 0.05 ^
ABA	245.0 ± 19.3	5.13 ± 3.97 &#*	5.49 ± 7.15	237 ± 22.2	260 ± 19.1	10.43 ± 21.61 &*

**Table 5:** HRV frequency-domain measures. Data expressed as mean ± SD. Statistically significant difference marked as: #—vs. RF, ^—vs. ABA.

	VLF Power (%)	LF Power (%)	LF Power (n.u.)	HF Power (%)	HF Power (n.u.)	LF/HF
C	24.90 ± 24.63	23.44 ± 23.02	28.62 ± 24.27	51.66 ± 24.02	71.38 ± 24.27	0.44 ± 0.53
RF	23.40 ± 17.26	7.85 ± 5.74	11.39 ± 10.64	68.75 ± 20.51 ^	88.61 ± 10.64	0.14 ± 0.16
Wh	41.60 ± 26.55	5.15 ± 2.37	9.75 ± 4.03	53.25 ± 24.95	90.25 ± 4.03	0.11 ± 0.05
ABA	45.50 ± 37.09	16.61 ± 14.94	32.37 ± 16.45	38.04 ± 30.20 #	67.63 ± 16.45	1.70 ± 3.26

**Table 6:** HRV nonlinear measures. Data expressed as mean ± SD. Statistically significant difference marked as: &—vs. C, #—vs. RF, *—vs. Wh, ^—vs. ABA.

	SD1	SD2	SD2/SD1	ApEn	SampEn	DFA1	sBP (mmHg)
C	3.27 ± 1.81	4.27 ± 2.11 ^	1.40 ± 0.62 ^	1.38 ± 0.21 ^	1.60 ± 0.38 ^	0.44 ± 0.19	91.32 ± 11.29
RF	3.16 ± 1.56	2.92 ± 0.73 ^	1.06 ± 0.35 ^	1.40 ± 0.18 ^	1.58 ± 0.39 ^	0.30 ± 0.17 ^	97.58 ± 29.36
Wh	2.3 ± 0.81	3.58 ± 1.74 ^	1.63 ± 0.88	1.39 ± 0.13 ^	1.54 ± 0.21 ^	0.32 ± 0.04 ^	105.95 ± 20.90
ABA	3.88 ± 5.03	5.75 ± 3.46 &#*	2.42 ± 1.79 &#	1.22 ± 0.22 &#*	1.29 ± 0.44 &#*	0.53 ± 0.22 #*	110.86 ± 14.32

## Data Availability

Data are available from authors upon reasonable request.

## References

[1] Routtenberg A., Kuznesof A.W. (1967). Self-starvation of rats living in activity wheels on a restricted feeding schedule. J. Comp. Physiol. Psychol..

[2] Rome E.S., Ammerman S. (2003). Medical complications of eating disorders: An update. J. Adolesc. Health.

[3] Olivares J.L., Vázquez M., Fleta J., Moreno L.A., Pérez-González J.M., Bueno M. (2005). Cardiac findings in adolescents with anorexia nervosa at diagnosis and after weight restoration. Eur. J. Pediatr..

[4] Sachs K.V., Harnke B., Mehler P.S., Krantz M.J. (2016). Cardiovascular complications of anorexia nervosa: A systematic review. Int. J. Eat. Disord..

[5] Arcelus J., Mitchell A.J., Wales J., Nielsen S. (2011). Mortality rates in patients with anorexia nervosa and other eating disorders: A meta-analysis of 36 studies. Arch. Gen. Psychiatry.

[6] Yager J., Devlin M.J., Halmi K.A., Herzog D.B., Mitchell J.E., Powers P., Zerbe K.J., McIntyre J.S., Charles S.C., Anzia D.J. (2006). Treatment of patients with eating disorders third edition. Am. J. Psychiatry.

[7] Aoki C., Avena N.M. (2021). Activity-based anorexia, an animal model of anorexia nervosa for investigating brain plasticity underlying the gain of resilience. Animal Models of Eating Disorders.

[8] Kurnik-Łucka M., Skowron K., Gil K., Avena N.M. (2021). In search for perfection: An activity-based rodent model of anorexia. Animal Models of Eating Disorders.

[9] Schalla M.A., Stengel A. (2019). Activity Based Anorexia as an Animal Model for Anorexia Nervosa—A Systematic Review. Front. Nutr..

[10] Termorshuizen J.D., Watson H.J., Thornton L.M., Borg S., Flatt R.E., MacDermod C.M., Harper L.E., van Furth E.F., Peat C.M., Bulik C.M. (2020). Early impact of COVID-19 on individuals with self-reported eating disorders: A survey of ~1000 individuals in the United States and the Netherlands. Int. J. Eat. Disord..

[11] Skowron K., Kurnik-łucka M., Dadański E., Bętkowska-Korpała B., Gil K. (2020). Backstage of eating disorder—About the biological mechanisms behind the symptoms of anorexia nervosa. Nutrients.

[12] Gutierrez E. (2013). A rat in the labyrinth of anorexia nervosa: Contributions of the activity-based anorexia rodent model to the understanding of anorexia nervosa. Int. J. Eat. Disord..

[13] Scherma M., Collu R., Satta V., Giunti E., Fadda P., Kobeissy F.H. (2019). Animal models of eating disorders. Psychiatric Disorders.

[14] Scharner S., Stengel A. (2021). Animal Models for Anorexia Nervosa—A Systematic Review. Front. Hum. Neurosci..

[15] Mondon C.E., Dolkas C.B., Sims C., Reaven G.M. (1985). Spontaneous running activity in male rats: Effect of age. J. Appl. Physiol..

[16] Perez-Leighton C.E., Grace M., Billington C.J., Kotz C.M. (2014). Role of spontaneous physical activity in prediction of susceptibility to activity based anorexia in male and female rats. Physiol. Behav..

[17] Pjetri E., de Haas R., de Jong S., Gelegen C., Oppelaar H., Verhagen L.A.W., Eijkemans M.J.C., Adan R.A., Olivier B., Kas M.J. (2012). Identifying Predictors of Activity Based Anorexia Susceptibility in Diverse Genetic Rodent Populations. PLoS ONE.

[18] Beneke W.M., Schulte S.E., Vander Tuig J.G. (1995). An analysis of excessive running in the development of activity anorexia. Physiol. Behav..

[19] Rosenfeld C.S. (2017). Sex-dependent differences in voluntary physical activity. J. Neurosci. Res..

[20] Edakubo S., Fushimi K. (2020). Mortality and risk assessment for anorexia nervosa in acute-care hospitals: A nationwide adminis-trative database analysis. BMC Psychiatry.

[21] Mitchison D., Mond J. (2015). Epidemiology of eating disorders, eating disordered behaviour, and body image disturbance in males: A narrative review. J. Eat. Disord..

[22] Ozawa Y., Shimizu T., Shishiba Y. (1998). Elevation of Serum Aminotransferase as a Sign of Multiorgan-Disorders in Severely Emaciated Anorexia Nervosa. Intern. Med..

[23] Ohwada R., Hotta M., Oikawa S., Takano K. (2006). Etiology of hypercholesterolemia in patients with anorexia nervosa. Int. J. Eat. Disord..

[24] Rautou P.E., Cazals-Hatem D., Moreau R., Francoz C., Feldmann G., Lebrec D., Ogier-Denis É., Bedossa P., Valla D., Durand F. (2008). Acute Liver Cell Damage in Patients with Anorexia Nervosa: A Possible Role of Starvation-Induced Hepatocyte Au-tophagy. Gastroenterology.

[25] Kheloufi M., Boulanger C.M., Durand F., Rautou P.E. (2014). Liver Autophagy in Anorexia Nervosa and Acute Liver Injury. Biomed. Res. Int..

[26] Harris R.H., Sasson G., Mehler P.S. (2013). Elevation of liver function tests in severe anorexianervosa. Int. J. Eat. Disord..

[27] Shephard R.J., Johnson N. (2015). Effects of physical activity upon the liver. Eur. J. Appl. Physiol..

[28] Hussain A.A., Hübel C., Hindborg M., Lindkvist E., Kastrup A.M., Yilmaz Z., Støving R.K., Bulik C.M., Sjögren J.M. (2019). Increased lipid and lipoprotein concentrations in anorexia nervosa: A systematic review and meta-analysis. Int. J. Eat. Disord..

[29] Misra M., Klibanski A. (2011). The neuroendocrine basis of anorexia nervosa and its impact on bone metabolism. Neuroendocrinology.

[30] Lai K.Y.C., De Bruyn R., Lask B., Bryant-Waugh R., Hankins M. (1994). Use of pelvic ultrasound to monitor ovarian and uterine maturity in childhood onset anorexia nervosa. Arch. Dis. Child..

[31] American Psychiatric Association (2013). DSM-5 Diagnostic Classification. Diagnostic and Statistical Manual of Mental Disorders.

[32] Levine R.L. (2002). Endocrine aspects of eating disorders in adolescents. Adolesc. Med..

[33] Cooke R.A., Chambers J.B., Singh R., Todd G.J., Smeeton N.C., Treasure J., Treasure T. (1994). QT interval in anorexia nervosa. Br. Heart J..

[34] Sall H., Timperley J. (2015). Bradycardia in anorexia nervosa. BMJ Case Rep..

[35] Panagiotopoulos C., McCrindle B.W., Hick K., Katzman D.K. (2000). Electrocardiographic findings in adolescents with eating disorders. Pediatrics.

[36] Vanderdonckt O., Lambert M., Cornejo Montero M., Boland B., Brohet C. (2001). The 12-lead electrocardiogram in anorexia nervosa: A report of 2 cases followed by a retrospective study. J. Electrocardiol..

[37] Dec G.W., Biederman J., Hougen T.J. (1987). Cardiovascular findings in adolescent inpatients with anorexia nervosa. Psychosom. Med..

[38] Facchini M., Sala L., Malfatto G., Bragato R., Redaelli G., Invitti C. (2006). Low-K+ dependent QT prolongation and risk for ventricular arrhythmia in anorexia nervosa. Int. J. Cardiol..

[39] Ravaldi C., Vannacci A., Ricca V. (2003). Complicanze cardiache dell’anoressia nervosa. Recenti Prog. Med..

[40] Peyser D., Scolnick B., Hildebrandt T., Taylor J.A. (2021). Heart rate variability as a biomarker for anorexia nervosa: A review. Eur. Eat. Disord. Rev..

[41] Lechin F., Van Der Dijs B., Pardey-Maldonado B., Rivera J.E., Baez S., Lechin M.E. (2010). Anorexia nervosa depends on adrenal sympathetic hyperactivity: Opposite neuroautonomic profile of hyperinsulinism syndrome. Diabetes Metab. Syndr. Obes. Targets Ther..

[42] Svorc P., Bačová I., Svorc P., Bužga M. (2013). Autonomic nervous system under ketamine/xylazine and pentobarbital anaesthesia in a Wistar rat model: A chronobiological view. Prague Med. Rep..

[43] Sekaninova N., Olexova L.B., Visnovcova Z., Ondrejka I., Tonhajzerova I. (2020). Role of Neuroendocrine, Immune, and Auto-nomic Nervous System in Anorexia Nervosa-Linked Cardiovascular Diseases. Int. J. Mol. Sci..

[44] Sirufo M.M., Ginaldi L., Martinis M. (2021). De Peripheral Vascular Abnormalities in Anorexia Nervosa: A Psycho-Neuro-Immune-Metabolic Connection. Int. J. Mol. Sci..

[45] Lipsman N., Woodside D.B., Lozano A.M. (2015). Neurocircuitry of limbic dysfunction in anorexia nervosa. Cortex.

[46] Monteleone A.M., Castellini G., Volpe U., Ricca V., Lelli L., Monteleone P., Maj M. (2018). Neuroendocrinology and brain imaging of reward in eating disorders: A possible key to the treatment of anorexia nervosa and bulimia nervosa. Prog. Neuro-Psychopharmacol. Biol. Psychiatry.

[47] Friederich H.C., Wu M., Simon J.J., Herzog W. (2013). Neurocircuit function in eating disorders. Int. J. Eat. Disord..

[48] Avena N.M., Bocarsly M.E. (2012). Dysregulation of brain reward systems in eating disorders: Neurochemical information from animal models of binge eating, bulimia nervosa, and anorexia nervosa. Neuropharmacology.

[49] Mottarlini F., Bottan G., Tarenzi B., Colciago A., Fumagalli F., Caffino L. (2020). Activity-based anorexia dynamically dysregulates the glutamatergic synapse in the nucleus accumbens of female adolescent rats. Nutrients.

[50] Skowron K., Jasiński K., Kurnik-Łucka M., Stach P., Kalita K., Węglarz W.P., Gil K. (2020). Hypothalamic and brain stem neu-rochemical profile in anorectic rats after peripheral administration of kisspeptin-10 using 1H-nmr spectroscopy in vivo. NMR Biomed..

[51] Méquinion M., Caron E., Zgheib S., Stievenard A., Zizzari P., Tolle V., Cortet B., Lucas S., Prévot V., Chauveau C. (2015). Physical activity: Benefit or weakness in metabolic adaptations in a mouse model of chronic food restriction?. Am. J. Physiol. Endocrinol. Metab..

[52] Hebebrand J., Exner C., Holtkamp C., Casper R., Remschmidt H., Herpertz-Dahlmann B., Klingenspor M. (2003). Hyperactivity in patients with anorexia nervosa and in semistarved rats: Evidence for a pivotal role of hypoleptinemia. Physiol. Behav..

